# Burden of physical inactivity and hospitalization costs due to chronic diseases

**DOI:** 10.1590/S0034-8910.2015049005650

**Published:** 2015-10-05

**Authors:** Renata Moraes Bielemann, Bruna Gonçalves Cordeiro da Silva, Carolina de Vargas Nunes Coll, Mariana Otero Xavier, Shana Ginar da Silva

**Affiliations:** I Programa de Pós-Graduação em Epidemiologia. Universidade Federal de Pelotas. Pelotas, RS, Brasil; II Faculdade de Nutrição. Universidade Federal de Pelotas. Pelotas, RS, Brasil

**Keywords:** Sedentary Lifestyle, Chronic Disease, Sickness Impact Profile, Hospitalization, economics, Unified Health System

## Abstract

**OBJECTIVE:**

To evaluate the physical inactivity-related inpatient costs of chronic non-communicable diseases.

**METHODS:**

This study used data from 2013, from Brazilian Unified Health System, regarding inpatient numbers and costs due to malignant colon and breast neoplasms, cerebrovascular diseases, ischemic heart diseases, hypertension, diabetes, and osteoporosis. In order to calculate the share physical inactivity represents in that, the physical inactivity-related risks, which apply to each disease, were considered, and physical inactivity prevalence during leisure activities was obtained from *Pesquisa Nacional por Amostra de Domicílio *(Brazil’s National Household Sample Survey). The analysis was stratified by genders and residing country regions of subjects who were 40 years or older. The physical inactivity-related hospitalization cost regarding each cause was multiplied by the respective share it regarded to.

**RESULTS:**

In 2013, 974,641 patients were admitted due to seven different causes in Brazil, which represented a high cost. South region was found to have the highest patient admission rate in most studied causes. The highest prevalences for physical inactivity were observed in North and Northeast regions. The highest inactivity-related share in men was found for osteoporosis in all regions (≈ 35.0%), whereas diabetes was found to have a higher share regarding inactivity in women (33.0% to 37.0% variation in the regions). Ischemic heart diseases accounted for the highest total costs that could be linked to physical inactivity in all regions and for both genders, being followed by cerebrovascular diseases. Approximately 15.0% of inpatient costs from Brazilian Unified Health System were connected to physical inactivity.

**CONCLUSIONS:**

Physical inactivity significantly impacts the number of patient admissions due to the evaluated causes and through their resulting costs, with different genders and country regions representing different shares.

## INTRODUCTION

Chronic non-communicable diseases became health care priorities due to their impact in morbidity and mortality and in the health care-related costs. Such disease burden is primarily felt in medium or low-income countries.^7^ In 2007, around 70.0% of deaths in Brazil were linked to chronic non-communicable diseases, considering the most prevalent morbidity causes in Brazil.^17^

As a consequence, such diseases are responsible for increased expenditures by Brazilian Unified Health System (SUS). According to data from the Ministry of Health, among the expenditures with Hospital Admission Authorizations (except births) in 2005, 58.0% of them concerned chronic diseases.^4^

Even though there is a relevant genetic component in the determination of those diseases, most of them can be prevented through life style changes, such as practice of physical activity.^2^ Nevertheless, even though the benefits from that practice are widely established in the literature,^2^ a low percentage of physically-active adults is observed in Brazil.^a^ A population survey with adult men and women from all capitals in Brazil, in 2012, showed that 33.5% of the adult population reached the recommended physical activity level. Another study pointed out that 10.5% of Brazilian subjects over 15 years of age or older, in 2008, were considered to be active during leisure activities.^11^

Lee et al^12^ evaluated the effects from physical inactivity in the burdens of chronic non-communicable diseases worldwide, and they approximately found a 6.0% disease burden for coronary heart disease, 7.0% for type 2 diabetes, 10.0% for breast cancer, and 10.0% for colon cancer. The same study found that over 533,000 and 1.3 million deaths could be avoided should physical inactivity be reduced by 10.0% or 25.0%, respectively. Other studies show the benefits from reducing physical inactivity in the financial costs which are related to health care.

This study aimed to evaluate the physical inactivity-related inpatient costs of chronic non-communicable diseases.

## METHODS

This consists of a descriptive study, which was conducted based on secondary data. This study used data from 2013, from SUS, regarding inpatient costs due to cancer, circulatory system diseases, diabetes, and osteoporosis. Regarding cancer, inpatient data and their respective costs were extracted, regarding malignant breast and colon neoplasms. Regarding the circulatory system, the data were separately obtained for cerebrovascular diseases, ischemic heart diseases, and hypertension.

Inpatient data and costs were obtained from Departamento *de Informática do SUS* (DATASUS – SUS’ Information Technology Department). The total number of 40-year or older inpatients and their total costs were identified by ages and regions of residence in Brazil (North, Northeast, Southeast, South, and Midwest). The data for studied causes were extracted from DATASUS, according to the latest International Statistical Classification of Diseases and Related Health Problems 10^th^ Revision (ICD-10). Regarding chapter II of ICD-10, information extracted concerned information on malignant colon neoplasms and malignant breast neoplasms. Regarding circulatory system diseases (chapter IX), information regarding ‘cerebrovascular diseases’ was extracted based on the causes for intracerebral hemorrhaging, cerebral infarction, cerebrovascular accidents that are not specified as hemorrhagic or ischemic, and other cerebrovascular diseases. Data were extracted regarding acute myocardial infarctions, other ischemic heart diseases, atherosclerosis, and heart failure for ischemic heart diseases.

The data on hypertension were extracted from cause primary hypertension. The inpatient data regarding diabetes mellitus (chapter IV) and osteoporosis (chapter XII) were also analyzed.

An inactivity-related fraction (IRF) was used. It aimed to identify the percentage reduction in the incidence of the disease in case patients were physically active. The inactivity-related fraction was calculated through the use of the following formula (p = exposure prevalence; RR - exposure-related relative risk):


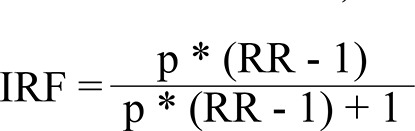


The RR for each of the seven hospital admission causes were separately obtained for men and women, from recent meta-analyses (Table 1). The choice of the related relative risks (RR) is justified through the use of some of them in a recent study published by Lee et al^12^ and through the availability of results that had been adjusted to known confounding factors in good methodological qualify and scientific impact articles. The study by Wolin et al,^19^ who respectively found RR of 1.32 e 1.27 for men and women, was used as a frame of reference for malignant colon neoplasms. The analysis of costs related to malignant breast neoplasms was only conducted for women, with an RR of 1.33 for calculating IRF, according to Monninkhof et al.^15^ For circulatory system diseases, we used the relative risks that were found by Li and Siegrist^13^ for cerebrovascular and ischemic heart diseases, the RR of 1.37 and 1.12 for cerebrovascular diseases and the RR of 1.18 and 1.28 for Ischemic heart diseases, respectively for men and women. The RR of physical inactivity regarding arterial hypertension was obtained from Huai et al,^8^ which described an inactivity-related RR of 1.12 in a single analysis including men and women. Values of 1.30 and 1.72 were respectively used for men and women regarding diabetes (Jeon et al^9^). The RR of 1.82 and 1.61 were used to respectively calculate IRF for femoral fractures for men and women, as described by Moayyeri^14^ regarding osteoporosis.

**Table 1:** Prevalence of physical inactivity and relative risks for diseases selected in adult people who are older than 40 years of age, physical inactivity-related fraction according to its causes. Regions in Brazil, 2013.

Variable	Relative risk		Physical inactivity-related fraction (%)									
			North		Northeast		Southeast		South		Midwest	
	Men	Women	Men	Women	Men	Women	Men	Women	Men	Women	Men	Women
Prevalence of physical activity (%)			79.9	80.8	79.9	77.0	73.9	73.7	70.5	68.5	74.3	70.0
Breast neoplasm^a^		1.33		21.1		20.3		19.6		18.4		18.8
Colon neoplasm	1.32	1.27	20.4	17.9	20.4	17.2	19.1	16.6	18.4	15.6	19.2	15.9
Cerebrovascular diseases	1.37	1.12	22.8	8.8	22.8	8.5	21.5	8.1	20.7	7.6	21.6	7.7
Ischemic heart diseases	1.18	1.28	12.6	18.4	12.6	17.7	11.7	17.1	11.3	16.1	11.8	16.4
Hypertension^b^	1.12	1.12	8.7	8.8	8.7	8.5	8.1	8.1	7.8	7.6	8.2	7.7
Diabetes	1.30	1.72	19.3	36.8	19.3	35.7	18.1	34.7	17.5	33.0	18.2	33.5
Osteoporosis	1.82	1.61	39.6	33.0	39.6	32.0	37.7	31.0	36.6	29.5	37.9	29.9

a Only for women.

b Unavailable gender-stratified data.

The estimated presence of physical inactivity was obtained through data from *Pesquisa Nacional por Amostra de Domicílio *(PNAD – Brazil’s National Household Sample Survey) of 2008.^b^ The presence of physical inactivity during leisure activities (i.e., no physical inactivity during leisure activities from subjects who are 40 years or older) was obtained separately according to regions and genders. The estimated prevalences were obtained by considering the effect from the experiment design. The *svy* command was used in Stata statistical software package, version 12.0.

Data from PNAD 2008 were used,^b^ considering the leisure domain, once most most analyzed studies regarding extraction of RR values only used recreational physical activity a factor of exposure. A more conservative measurement was adopted, considering physical inactivity as the failure to perform any physical activity during leisure periods, disregarding subjects’ not meeting the minimum recommended period of 30 minutes a day. The use of that classification from PNAD,^b^ is justified because of the nation-wide availability of its probability sampling data. That was only possible through this investigation.

The hospital admission rates for each of the seven studied causes were calculated per individual regions. The denominator was the last estimate that was made available by Brazilian Institute of Geography and Statistics (IBGE)^c^ regarding individual genders and age ranges in the population, from July 1, 2012. Those data were used due to the need for identifying subjects from both genders who were 40 years or older according to their regions of residence, which was not possible with the estimate that had been published for 2013. The percentage share of IRF for each admission cause was obtained in a stratified manner, according to genders and regions. The costs with hospital admissions for each physical inactivity-related cause were multiplied by their respective IRF values.

## RESULTS

In 2003, 974,641 patients were admitted due to chronic diseases that were evaluated in adults who were 40 years or older in Brazil. Those admissions had a cost of R$1,848,627,410.03 (US$695.6 million)^d^ to SUS.

**Figure 1:**
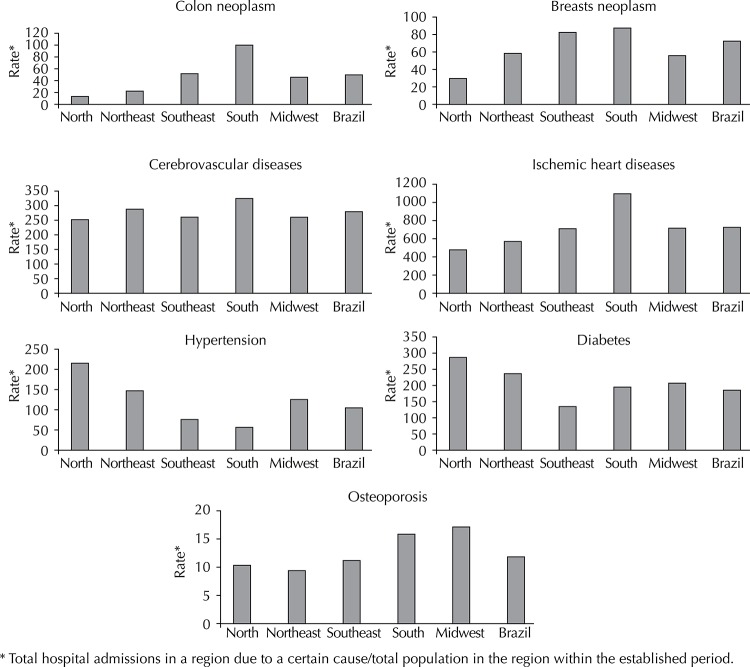
Rate of hospital admissions in Brazilian Unified Health System per 100,000 inhabitants, according to causes and regions in Brazil, 2013.

South region was observed to have the highest admission rate for five of the analyzed diseases (colon and breast neoplasms, cerebrovascular and ischemic heart diseases, and osteoporosis) (Figure 1). North region was observed to lead the hospital admission rate for diabetes and hypertension (287.5 and 221.3 hospital admissions/100,000 inhabitants, respectively). Among evaluated morbidities, the highest hospital admission rates were observed for ischemic heart diseases in all regions, with a higher rate in South region and a lower on in Brazil’s North region.

The highest prevalence for physical inactivity during leisure periods was observed in North and Northeast regions, and the lowest one was found to be in South region, for both genders (Table 1). Osteoporosis was the cause with the highest physical inactivity-related fraction in men for all regions (around 35.0%), followed by cerebrovascular diseases. Diabetes was the cause with the highest physical inactivity-related fraction in women. The pattern was similar for this group in all regions, from 33.0% (South region) to 37.0% (North region) of diabetes-related hospital admissions being linked to physical inactivity.

Ischemic heart diseases accounted for the highest total cost volume that could be linked to physical inactivity in all analyzed regions and for both genders, being followed by cerebrovascular diseases (Tables 2 and 3). The highest physical-inactivity related inpatient cost, when ischemic heart diseases are analyzed, was found for Southeast region (R$43,267,131.09 and R$39,273,314.16 – approximately US$16.3 and US$14.7 million dollars – for men and women, respectively). Over 20.0% of hospital admissions costs from cerebrovascular diseases in men were linked to physical inactivity – in turn, they accounted for around 8.0% in women.

**Table 2:** Total costs (R$) with hospital admissions related to physical inactivity in men who are 40 years or older, according to causes and regions. Brazil, 2013.

Variable	North		Northeast		Southeast		South		Midwest	
	Total cost of hospital admissions	Total cost of hospital admissions related to physical inactivity	Total cost of hospital admissions	Total cost of hospital admissions related to physical inactivity	Total cost of hospital admissions	Total cost of hospital admissions related to physical inactivity	Total cost of hospital admissions	Total cost of hospital admissions related to physical inactivity	Total cost of hospital admissions	Total cost of hospital admissions related to physical inactivity
Colon neoplasm	528,909.08	107,897.45	4,150,374.36	846,676.37	17,841,337.34	3,407,695.432	10,400,014.14	1,913,602.602	1,906,174.60	365,985.52
Cerebrovascular diseases	6,990,639.78	1,593,865.87	29,118,203.9	6,638,950.49	75,467,353.8	16,225,481.07	33,348,709.38	6,903,182.84	10,071,668.46	2,175,480.39
Ischemic heart diseases	25,807,466.73	3,251,740.81	123,113,499.15	15,512,300.89	369,804,539.27	43,267,131.09	208,104,992.17	23,515,864.12	54,451,256.33	6,425,248.25
Hypertension	1,107,994.55	97,503.52	4,600,485.53	391,041.27	5,880,692.57	476,336.1	988,858.94	75,153.279	846,169	65,155.01
Diabetes	2,833,554.92	546,876.10	9,101,133.13	1,756,518.69	12,522,594.86	2,266,589.67	5,352,859.72	936,750.45	2,454,322.32	446,686.66
Osteoporosis	365,122.81	144,588.63	1,530,036.63	605,894.51	3,172,116.60	1,195,887.96	1,344,026.02	491,913.52	592,409.51	224,523.20

**Table 3:** Total costs (R$) with hospital admissions related to physical inactivity in women who are 40 years or older, according to causes and regions. Brazil, 2013.

Variable	Physical inactivity-related fraction (%)									
	North		Northeast		Southeast		South		Midwest	
	Total cost of hospital admissions	Total cost of hospital admissions related to physical inactivity	Total cost of hospital admissions	Total cost of hospital admissions related to physical inactivity	Total cost of hospital admissions	Total cost of hospital admissions related to physical inactivity	Total cost of hospital admissions	Total cost of hospital admissions related to physical inactivity	Total cost of hospital admissions	Total cost of hospital admissions related to physical inactivity
Breast neoplasm	1,932,885.18	407,838.77	22,554,041.46	4,578,470.42	42,193,619.91	8,269,949.50	15,388,384.93	2,831,462.83	4,010,343.4	753,944.56
Colon neoplasm	565,459.13	101,217.18	5,417,881.97	931,875.70	18,120,101.18	3,007,936.80	9,278,149.77	1,447,391.36	1,797,318.95	285,773.71
Cerebrovascular disease	7,545,551.11	664,008.50	34,431,850.96	2,926,707.33	74,977,064.66	6,073,142.24	34,944,666.69	2,655,794.67	9,342,646.7	719,383.80
Ischemic heart disease	12,968,545.40	2,386,212.35	87,039,346.79	15,405,964.38	229,668,503.85	39,273,314.16	129,686,406.57	20,879,511.46	31,308,312.37	5,134,563.23
Hypertension	857,181.07	74,574.75	2,879,346.81	250,503.17	5,464,663.57	442,637.75	679,199.82	52,977.59	611,939.48	50,179.04
Diabetes	3,310,500.49	1,218,264.18	11,879,611.2	4,241,021.20	12,716,804.35	4,412,731.11	6,340,251.14	2,092,282.88	2,568,922.73	860,589.11
Osteoporosis	220,709.64	72,834.18	809,548.22	259,055.43	2,112,057.67	654,737.88	927,609.97	273,644.94	280,467.29	83,859.72

**Figure 2:**
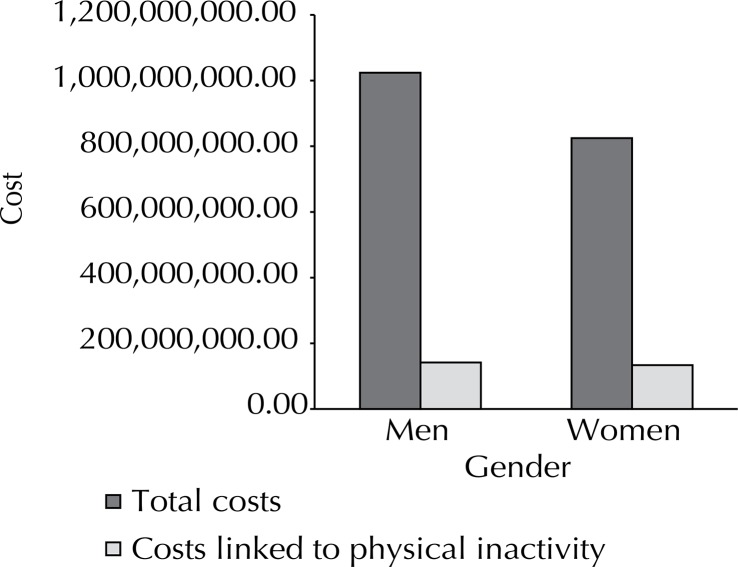
Total costs (R$) to Brazilian Unified Health System due to hospital admissions that were caused by colon and breast neoplasms, ischemic heart and cerebrovascular diseases, hypertension, diabetes, and osteoporosis, and physical inactivity-related value for men and women. Brazil, 2013.

For men, R$1,023,797,515.60 (US$2,720,946,657.21) were spent with hospital admissions due to those diseases. Among those, R$141,872,521.76 (US$377,054,601.08) were linked to physical inactivity (14.0%). The total cost for female inpatients was R$824,829,894.43 (US$2,192,150,410.43) – 16.2% of that amount was linked to physical inactivity (Figure 2).

## DISCUSSION

Out of total hospital admissions at SUS in 2013, 15.0% of them were linked to physical inactivity, which led to a total estimated cost of R$275,646,877.64 (US$732,586,706.70). Hospital admissions due to osteoporosis were the ones with the highest physical inactivity-related fraction in all regions of the country (approximately 40.0%) for men; for women, diabetes-related admissions accounted for the highest fraction (approximately 35.0%). Besides that, ischemic heart diseases accounted for the highest total share of the total cost of hospital admissions that could be linked to physical inactivity for both genders and in all regions.

Studies on the economic impact of risk factors in Brazil’s public health care service are scarce. A study that was conducted by Pinto and Ugá^16^ in 2005 found that the smoking-related costs of hospital admissions due to ischemic heart diseases and cerebrovascular diseases respectively made up for shares of approximately 20.0% and 22.0% of total costs for those diseases. In this study, the percentage of physical inactivity-related costs of hospital admissions due to both diseases was approximately 15.0%.

Sichieri et al^18^ analyzed the overweight and obesity-related hospital admission costs in 20 to 60-year old patients in 2001, and they observed that diabetes-related admissions had the highest overweight and obesity-related fractions (39.0% and 34.0%, respectively). In this study, 26.6% diabetes-related hospital admissions were linked to physical inactivity.

Bielemann et al^3^ estimated that costs could be reduced if inpatients suffering from circulatory system diseases and diabetes were encouraged to practice physical activity – Pelotas, RS, Southern Brazil, 2007. There would be a 50.0% economic impact from the reduction in inpatient costs due to circulatory system diseases, and a 13.0% one in diabetes-related hospital admission costs if the whole population became physically active.

A study by Rezende et al^6^ evaluated the physical inactivity impact on morbidity and mortality rates due to four chronic non-communicable diseases (cardiovascular diseases, type 2 diabetes, breast cancer, colon cancer) in Brazil, and found that physical inactivity accounted for 3.0% to 5.0% of the incidence rates of those diseases, and for 5.3% of mortality rates due to all causes. Such results reinforce the role of physical inactivity as an important risk factor, causing a significant impact in the burden from chronic diseases.^6^

Studies have documented a significant economic impact from physical inactivity in health care systems worldwide. A study that was conducted in Canada found that 2.5% of total medical expenses with chronic diseases in 1999 regarded to physical inactivity.^10^ For the early 1990s United Kingdom, 6.5 billion pounds were estimated to be spent in the health care system, 16.0% of which (1.06 pounds – approximately R$4.4 billion and US$1.7 billion) were allegedly spent due to physical inactivity in the early 2000s,^1^ a percentage which is similar to the one found in this study results. In China, physical inactivity accounts for over 15.0% of medical or non-medical expenses that are associated with the main chronic diseases in the country.^20^ In Australia, the 10.0% reduction in physical inactivity would result in 6,000 fewer disease cases a year, and 2,000 fewer deaths, which would result in a great economic benefit.^5^

With a 10.0% reduction in the prevalence of physical inactivity in the Brazilian population (from 74.5% to 67.05%), costs would be reduced by R$24,081,636.89 (US$64,001,766.36) with hospital admissions due to studied causes. The economic benefit would correspond to an approximate 1.3% reduction in the total cost of admissions at SUS due to analyzed causes. However, comparison between these results and the ones from other studies can only be conducted in a limited way, as different methodologies, populations, and periods were used to estimate the economic impact from physical inactivity. In addition to that, one has to take into account the structure differences in health care systems among countries.

A possible limitation for this study lies in the fact that the RR which were used to estimate the related fraction were taken off the literature and arise from studies that were conducted in developed countries, with populations that are different from the Brazilian one. The quality of supervision systems in Brazil may be another limitation. Even though the quality of the information that is generated by the Hospital Information System has improved over the last years, the quality of the system is far from being ideal due to the low-quality information that is provided in *Autorizações de Internação Hospitalares* (Hospital Admission Authorization) forms.^e^ ^,^ ^f^ Due to that, a great deal of hospital admissions due to osteoporotic fractures may have been attributed to cause S72 of ICD-10 – femoral fracture – which results in the underestimation of values shown. Nevertheless, a more conservative measure was chosen to be adopted, instead of analyzing hospital admissions whose causes were considered as femoral fractures, which would lead hospital admissions and related costs to be overestimated. Lastly, the prevalence of physical inactivity that was used (zero minutes/week as of PNAD) may have overestimated the costs which can be attributed to physical inactivity in the admissions by SUS, once IRF calculation depends on the prevalence of the factor of exposure. However, the use of zero minutes/week was preferred, as the physical inactivity classification that was used by different studies provides the RR values that enable IRF calculation. That prevents discrepancies from occurring among other possible thresholds (e.g., 30 minutes/week).

The results in this study are concluded to significantly contribute to assessing the impact from physical inactivity in Brazil’s health care system. Calculating the inactivity-related fraction according to genders and regions in the country is important, as it considers the heterogeneity between men and women, and also the epidemiological profiles of regions. It also allows for more reliable identification of the impact from physical inactivity to publicly-funded hospital admissions.

Physical inactivity significantly impacts the number of patient admissions due to the evaluated chronic diseases and their resulting costs, with different genders and country regions representing different shares in costs which arise from inactivity, depending on genders and regions in the country. Thus, further investment on intervention strategies that aim to reduce physical inactivity are necessary, as it would contribute to expressively reducing the expenditures in the public health care system and improving the quality of life and health conditions of the Brazilian population.

## Footnotes

c Instituto Brasileiro de Geografia e Estatística. Estimativas populacionais para os municípios brasileiros em 1/7/2012. [cited 2015 Aug 3]. Available from: http://www.ibge.gov.br/home/estatistica/populacao/estimativa2012/default.shtm

d Exchange rate for conversion in American dollars: R$2.6577 as of December 31, 2014. [cited 2015 Aug 3]. Available from: http://pt.exchange-rates.org/Rate/USD/BRL/31-12-2014

e Pinto R. Sistemas de informações hospitalares de Brasil, Espanha e Portugal: semelhanças e diferenças [dissertation]. Rio de Janeiro (RJ): Fundação Oswaldo Cruz, Escola Nacional de Saúde Pública Sérgio Arouca; 2010 [cited 2015 Apr 29]. Available from: http://www.arca.fiocruz.br/bitstream/icict/2292/1/ENSP_Disserta%C3%A7%C3%A3o_Pinto_Rosana.pdf

f Santos A. Sistema de Informações Hospitalares do Sistema Único de Saúde: documentação do sistema para auxiliar o uso das suas informações [dissertation]. Rio de Janeiro (RJ): Fundação Oswaldo Cruz, Escola Nacional de Saúde Pública Sérgio Arouca; 2009 [cited 2015 Apr 29]. Available from: http://www.arca.fiocruz.br/bitstream/icict/2372/1/ENSP_Disserta%C3%A7%C3%A3o_Santos_Andr%C3%A9ia_Cristina.pdf
