# Supplementary material for: Burden of physical inactivity and hospitalization costs due to chronic diseases
Source: Rev Saude Publica. 2015 Oct 5;49:75. doi: 10.1590/S0034-8910.2015049005650 (PMC4603264; doi:10.1590/S0034-8910.2015049005650)
Supplement: Translation

# Translation: Impacto da inatividade física e custos de hospitalização por doenças crônicas

**Authors:** Renata Moraes Bielemann, Bruna Gonçalves Cordeiro da Silva, Carolina de Vargas Nunes Coll, Mariana Otero Xavier, Shana Ginar da Silva

**Affiliations:** I Programa de Pós-Graduação em Epidemiologia. Universidade Federal de Pelotas. Pelotas, RS, Brasil; II Faculdade de Nutrição. Universidade Federal de Pelotas. Pelotas, RS, Brasil

**Keywords:** Estilo de Vida Sedentário, Doença Crônica, Perfil de Impacto da Doença, Hospitalização, economia, Sistema Único de Saúde

## Abstract

**OBJETIVO:**

Avaliar o custo de internações por doenças crônicas não transmissíveis atribuível à inatividade física.

**MÉTODOS:**

Este estudo utilizou dados de 2013, do Sistema Único de Saúde, referentes ao número e respectivo custo das internações por neoplasia maligna de cólon e mama, doenças cerebrovasculares, doenças isquêmicas do coração, hipertensão, diabetes e osteoporose. Para o cálculo da fração atribuível à inatividade física foram considerados os riscos relativos da inatividade física a cada doença e a prevalência de inatividade física no lazer foi obtida da Pesquisa Nacional por Amostra de Domicílio. A análise foi estratificada por sexo e região do País de indivíduos com idade igual ou superior a 40 anos. O custo das internações de cada causa atribuível à inatividade física foi multiplicado pela respectiva fração a ela atribuível.

**RESULTADOS:**

Foram realizadas 974.641 internações hospitalares por sete causas de internações no Brasil, em 2013, o que representou custo alto. A região Sul apresentou a maior taxa de internação na maioria das causas estudadas. A maior prevalência de inatividade física ocorreu nas regiões Norte e Nordeste. A maior fração atribuível à inatividade em homens foi encontrada para a osteoporose em todas as regiões (≈ 35,0%), enquanto o diabetes apresentou maior fração atribuível à inatividade em mulheres (variação de 33,0% a 37,0% nas regiões). As doenças isquêmicas do coração foram responsáveis pelos mais altos custos totais e atribuíveis à inatividade física em todas as regiões e em ambos os sexos, seguidas das doenças cerebrovasculares. Aproximadamente 15,0% dos custos ao Sistema Único de Saúde das internações foi atribuível à inatividade física.

**CONCLUSÕES:**

A inatividade física impacta significativamente o número de internações hospitalares pelas causas avaliadas e nos custos resultantes, com diferenças na ocorrência dependendo do sexo e região do País.

## INTRODUÇÃO

As doenças crônicas não transmissíveis tornaram-se prioridades em saúde devido ao seu impacto na morbimortalidade e nos custos decorrentes de assistência médica. A carga dessas doenças recai principalmente sobre países de renda média ou baixa.^7^ Em 2007, cerca de 70,0% das mortes no Brasil foram atribuídas às doenças crônicas não transmissíveis, consideradas as morbidades mais prevalentes no País.^17^

Como consequência, tais doenças são responsáveis pelos maiores gastos no Sistema Único de Saúde (SUS). Segundo dados do Ministério da Saúde, dos gastos com pagamento de Autorizações de Internação Hospitalar (exceto partos) em 2005, 58,0% foram atribuídos às doenças crônicas.^4^

Ainda que haja um componente genético relevante na determinação da suscetibilidade a essas doenças, a maior parte delas pode ser prevenida mediante alterações no estilo de vida, como a prática de atividade física.^2^ No entanto, ainda que os benefícios decorrentes dessa prática estejam amplamente estabelecidos na literatura,^2^ observa-se baixo percentual de adultos fisicamente ativos no Brasil.^a^ Inquérito populacional com homens e mulheres adultos de todas as capitais do País, em 2012, mostrou que 33,5% da população adulta atingiu o nível recomendado de atividade física. Outra pesquisa apontou que 10,5% dos indivíduos brasileiros com 15 anos ou mais de idade, em 2008, foram considerados ativos no lazer.^11^

Lee et al^12^ avaliaram os efeitos da inatividade física na carga de doenças crônicas não transmissíveis no mundo e encontraram aproximadamente 6,0% da carga de doença para doença coronariana, 7,0% para diabetes tipo 2, 10,0% para câncer de mama e 10,0% para câncer de colo. O mesmo estudo encontrou que mais de 533.000 e 1,3 milhão de mortes poderiam ser evitadas caso a inatividade física diminuísse em 10,0% ou 25,0%, respectivamente. Outros estudos mostram os benefícios da redução da inatividade física nos custos financeiros relacionados à assistência à saúde.

O objetivo do presente estudo foi avaliar o custo das internações por doenças crônicas não transmissíveis atribuível à inatividade física.

## MÉTODOS

Estudo descritivo, realizado a partir de dados secundários. Foram examinados dados de 2013, do Sistema Único de Saúde, referentes ao custo das internações por câncer, doenças do aparelho circulatório, diabetes e osteoporose. Com relação ao câncer, foram extraídos dados de internação e respectivos custos devidos a neoplasias malignas de mama e cólon. Para o aparelho circulatório, foram obtidos, separadamente, os dados para doenças cerebrovasculares, doenças isquêmicas do coração e hipertensão.

Os dados de internações e seus custos foram obtidos do Departamento de Informática do SUS (Datasus). Foram identificados o número total de internações e seu custo total em indivíduos de 40 anos ou mais por sexo e região do País (Norte, Nordeste, Sudeste, Sul e Centro-Oeste). Os dados para as causas estudadas foram extraídos no Datasus, conforme última Classificação Internacional das Doenças, 10ª Revisão (CID-10). No que tange ao capítulo II da CID10, foram extraídas informações de Neoplasia Maligna do Cólon e Neoplasia Maligna de Mama. Quanto às Doenças do Aparelho Circulatório (capítulo IX), as informações acerca das ‘doenças cerebrovasculares’ foram extraídas com base nas causas de Hemorragia Intracerebral, Infarto Cerebral, Acidente Vascular Cerebral Não-Especificado como hemorrágico ou isquêmico e Outras Doenças Cerebrovasculares. Foram extraídos dados para Infarto Agudo do Miocárdio, Outras Doenças Isquêmicas do Coração, Aterosclerose e Insuficiência Cardíaca para doenças isquêmicas do coração.

Os dados sobre hipertensão foram extraídos da causa Hipertensão Essencial (primária). Foram também analisados dados provenientes das internações causadas pelo Diabetes Mellitus (capítulo IV) e Osteoporose (capítulo XIII).

Utilizou-se a fração atribuível à inatividade (FAI), que visa identificar a redução percentual na incidência de doença caso a inatividade física não estivesse presente. O cálculo da fração atribuível à inatividade deu-se mediante utilização da seguinte fórmula (p = prevalência de exposição; RR = risco relativo atribuído à exposição):



Os RR para cada uma das sete causas de internações foram obtidos a partir de meta-análises recentes, separadamente para homens e mulheres (Tabela 1). A escolha dos respectivos riscos relativos (RR) justifica-se pela utilização de alguns deles, em estudo recente, publicado por Lee et al^12^ e pela disponibilidade de resultados ajustados a fatores de confusão conhecidos em artigos de boa qualidade metodológica e impacto científico. Para a neoplasia maligna de cólon, utilizou-se referência de Wolin et al^19^ que encontrou RR de 1,32 e 1,27 para homens e mulheres, respectivamente. A análise dos custos relacionados à neoplasia maligna de mama foi conduzida apenas para mulheres, com RR de 1,33 para cálculo da FAI, conforme Monninkhof et al.^15^ Para doenças do aparelho circulatório, utilizamos os riscos relativos encontrados por Li e Siegrist^13^ para doenças cerebrovasculares e isquêmicas do coração, os RR de 1,37 e 1,12 para doenças cerebrovasculares e de 1,18 e 1,28 para doenças isquêmicas do coração, respectivamente para homens e mulheres. O RR da inatividade física para a ocorrência de hipertensão arterial foi obtido de Huai et al,^8^ que descreveu RR da inatividade de 1,12 em análise única incluindo homens e mulheres. Para o diabetes, foi utilizado como RR os valores de 1,30 para homens e 1,72 para mulheres (Jeon et al^9^). Para o cálculo da FAI para fratura do fêmur, utilizou-se o RR de 1,82 e 1,61 para homens e mulheres, respectivamente, descrito por Moayyeri^14^ em relação à ocorrência de osteoporose.

**Tabela 1:** Prevalência de inatividade física e riscos relativos para as doenças selecionadas em adultos maiores de 40 anos e fração atribuível à inatividade física, segundo causa. Regiões do Brasil, 2013.

Variável	Risco relativo		Fração atribuível à inatividade física (%)									
			Norte		Nordeste		Sudeste		Sul		Centro-Oeste	
	Homens	Mulheres	Homens	Mulheres	Homens	Mulheres	Homens	Mulheres	Homens	Mulheres	Homens	Mulheres
Prevalência de inatividade física (%)			79,9	80,8	79,9	77,0	73,9	73,7	70,5	68,5	74,3	70,0
Neoplasia de mama^a^		1,33		21,1		20,3		19,6		18,4		18,8
Neoplasia de cólon	1,32	1,27	20,4	17,9	20,4	17,2	19,1	16,6	18,4	15,6	19,2	15,9
Doenças cerebrovasculares	1,37	1,12	22,8	8,8	22,8	8,5	21,5	8,1	20,7	7,6	21,6	7,7
Doenças isquêmicas do coração	1,18	1,28	12,6	18,4	12,6	17,7	11,7	17,1	11,3	16,1	11,8	16,4
Hipertensão^b^	1,12	1,12	8,7	8,8	8,7	8,5	8,1	8,1	7,8	7,6	8,2	7,7
Diabetes	1,30	1,72	19,3	36,8	19,3	35,7	18,1	34,7	17,5	33,0	18,2	33,5
Osteoporose	1,82	1,61	39,6	33,0	39,6	32,0	37,7	31,0	36,6	29,5	37,9	29,9

a Somente para mulheres.

b Dados estratificados por sexo não disponíveis.

A estimativa da prevalência de inatividade física foi obtida por meio dos dados da Pesquisa Nacional por Amostra de Domicílios (PNAD) de 2008.^b^ Foi obtida a prevalência de inatividade física no lazer, i.e., nenhuma atividade física no lazer em indivíduos com idade igual ou superior a 40 anos, separadamente por região e sexo. As estimativas das prevalências foram obtidas considerando o efeito de delineamento amostral. Utilizou-se o comando *svy* no pacote estatístico Stata, versão 12.0.

Foram utilizados dados da PNAD 2008,^b^ considerando o domínio lazer, uma vez que a maior parte dos estudos analisados para extração dos valores de RR utilizou apenas a atividade física de lazer como exposição. Uma medida mais conservadora foi adotada considerando inatividade física a não realização de qualquer atividade física no período de lazer, desconsiderando o não alcance da recomendação de pelo menos 30 min/dia. A utilização dessa classificação, a partir da PNAD,^b^ justifica-se pela disponibilidade nacional dos dados por amostragem probabilística. Isso foi possível somente por meio desse inquérito.

As taxas de internações para cada uma das sete causas estudadas foram calculadas por região. O denominador foi a última estimativa disponibilizada pelo IBGE^c^ da população, segundo sexo e faixa etária, de 1^o^ de julho de 2012. Utilizaram-se esses dados pela necessidade da identificação de indivíduos com idade igual ou superior a 40 anos de ambos os sexos por região do País, o que não foi possível com a estimativa publicada para 2013. O valor percentual da FAI de cada causa de internação foi obtido de maneira estratificada por sexo e região. Os custos das internações de cada causa atribuível à inatividade física foram multiplicados pela respectiva FAI.

## RESULTADOS

Foram realizadas 974.641 internações hospitalares devido às doenças crônicas avaliadas em adultos com idade igual ou superior a 40 anos no Brasil, em 2013. Essas internações tiveram custo de R$1.848.627.410,03 (US$695,6 milhões)^d^ para o SUS.

A região Sul apresentou a maior taxa de internação em cinco das doenças analisadas (neoplasia de cólon e de mama, doenças cerebrovasculares e isquêmicas do coração e osteoporose) (Figura 1). A região Norte liderou a taxa de internações hospitalares para diabetes e hipertensão (287,5 e 221,3 internações/100.000 habitantes, respectivamente). Dentre as morbidades avaliadas, as maiores taxas de internação foram para as doenças isquêmicas do coração em todas as regiões, com taxa mais elevada na região Sul e mais baixa na região Norte do País.

**Figura 1:** Taxa de internações no Sistema Único de Saúde por 100.000 habitantes, segundo causa e regiões do Brasil, 2013.

A maior prevalência de inatividade física no lazer foi observada nas regiões Norte e Nordeste e a mais baixa na região Sul, para ambos os sexos (Tabela 1). A osteoporose foi a causa que mostrou maior fração atribuível à inatividade física em homens em todas as regiões (em torno de 35,0%), seguida das doenças cerebrovasculares. O diabetes foi a causa com maior fração atribuível à inatividade física nas mulheres. O padrão foi similar para esse grupo, entre as regiões, variando de 33,0% (região Sul) e 37,0% (região Norte) das internações por diabetes foram atribuídas à inatividade física.

As doenças isquêmicas do coração foram responsáveis pelo maior volume de custos totais e atribuíveis à inatividade física em todas as regiões analisadas e em ambos os sexos, seguidas das doenças cerebrovasculares (Tabelas 2 e 3). O maior custo de internações associadas à inatividade física, quando analisadas as doenças isquêmicas do coração, foi encontrado para a região Sudeste (R$43.267.131,09 e R$39.273.314,16 – aproximadamente US$16,3 e US$14,7 milhões de dólares – para homens e mulheres, respectivamente). Mais de 20,0% dos custos das internações por doenças cerebrovasculares em homens foram atribuídos à inatividade física, enquanto representaram cerca de 8,0% em mulheres.

**Tabela 2:** Custos totais (R$) das internações e atribuíveis à inatividade física de homens com idade igual ou superior a 40 anos, segundo causa e regiões. Brasil, 2013.

Variável	Norte		Nordeste		Sudeste		Sul		Centro-Oeste	
	Custo total de internações	Custo total de internações atribuíveis à inatividade física	Custo total de internações	Custo total de internações atribuíveis à inatividade física	Custo total de internações	Custo total de internações atribuíveis à inatividade física	Custo total de internações	Custo total de internações atribuíveis à inatividade física	Custo total de internações	Custo total de internações atribuíveis à inatividade física
Neoplasia de cólon	528.909,08	107.897,45	4.150.374,36	846.676,37	17.841.337,34	3.407.695,432	10.400.014,14	1.913.602,602	1.906.174,60	365.985,52
Doenças cerebrovasculares	6.990.639,78	1.593.865,87	29.118.203,9	6.638.950,49	75.467.353,8	16.225.481,07	33.348.709,38	6.903.182,84	10.071.668,46	2.175.480,39
Doenças isquêmicas do coração	25.807.466,73	3.251.740,81	123.113.499,15	15.512.300,89	369.804.539,27	43.267.131,09	208.104.992,17	23.515.864,12	54.451.256,33	6.425.248,25
Hipertensão	1.107.994,55	97.503,52	4.600.485,53	391.041,27	5.880.692,57	476.336,1	988.858,94	75.153,279	846.169	65.155,01
Diabetes	2.833.554,92	546.876,10	9.101.133,13	1.756.518,69	12.522.594,86	2.266.589,67	5.352.859,72	936.750,45	2.454.322,32	446.686,66
Osteoporose	365.122,81	144.588,63	1.530.036,63	605.894,51	3.172.116,60	1.195.887,96	1.344.026,02	491.913,52	592.409,51	224.523,20

**Tabela 3:** Custos totais (R$) das internações e atribuíveis à inatividade física em mulheres de idade igual ou superior a 40 anos, segundo causa e regiões. Brasil, 2013.

Variável	Fração atribuível à inatividade física									
	Norte		Nordeste		Sudeste		Sul		Centro-Oeste	
	Custo total de internações	Custo total de internações atribuíveis à inatividade física	Custo total de internações	Custo total de internações atribuíveis à inatividade física	Custo total de internações	Custo total de internações atribuíveis à inatividade física	Custo total de internações	Custo total de internações atribuíveis à inatividade física	Custo total de internações	Custo total de internações atribuíveis à inatividade física
Neoplasia de mama	1.932.885,18	407.838,77	22.554.041,46	4.578.470,42	42.193.619,91	8.269.949,50	15.388.384,93	2.831.462,83	4.010.343,4	753.944,56
Neoplasia de cólon	565.459,13	101.217,18	5.417.881,97	931.875,70	18.120.101,18	3.007.936,80	9.278.149,77	1.447.391,36	1.797.318,95	285.773,71
Doença cerebrovascular	7.545.551,11	664.008,50	34.431.850,96	2.926.707,33	74.977.064,66	6.073.142,24	34.944.666,69	2.655.794,67	9.342.646,7	719.383,80
Doença isquêmica do coração	12.968.545,40	2.386.212,35	87.039.346,79	15.405.964,38	229.668.503,85	39.273.314,16	129.686.406,57	20.879.511,46	31.308.312,37	5.134.563,23
Hipertensão	857.181,07	74.574,75	2.879.346,81	250.503,17	5.464.663,57	442.637,75	679.199,82	52.977,59	611.939,48	50.179,04
Diabetes	3.310.500,49	1.218.264,18	11.879.611,2	4.241.021,20	12.716.804,35	4.412.731,11	6.340.251,14	2.092.282,88	2.568.922,73	860.589,11
Osteoporose	220.709,64	72.834,18	809.548,22	259.055,43	2.112.057,67	654.737,88	927.609,97	273.644,94	280.467,29	83.859,72

Foram gastos R$1.023.797.515,60 (US$2.720.946.657,21) com internações hospitalares devido a essas doenças em homens. Destes, R$141.872.521,76 (US$377.054.601,08) foram atribuídos à inatividade física (14,0%). O custo total devido às internações de mulheres foi de R$824.829.894,43 (US$2.192.150.410,43) e 16,2% deste valor foram atribuídos à inatividade física (Figura 2).

**Figura 2:** Custos totais (R$) ao Sistema Único de Saúde devido às hospitalizações por neoplasia de cólon e mama, doenças isquêmicas do coração e cerebrovasculares, hipertensão, diabetes e osteoporose e valor atribuível à inatividade física de homens e mulheres. Brasil, 2013.

## DISCUSSÃO

Do total de internações em 2013, pelo SUS, 15,0% foram atribuídas à inatividade física, resultando em um custo total estimado em R$275.646.877,64 (US$732.586.706, 70). As internações por osteoporose foram aquelas com maior fração atribuível à inatividade física em todas as regiões do País (aproximadamente 40,0%) para os homens; para as mulheres, as internações por diabetes foram responsáveis pela maior fração atribuível (aproximadamente 35,0%). Além disso, as doenças isquêmicas do coração foram responsáveis pelos maiores gastos do custo total das internações atribuíveis à inatividade física para ambos os sexos e em todas as regiões.

Estudos sobre o impacto econômico de fatores de risco no sistema público de saúde são escassos no Brasil. Estudo conduzido por Pinto e Ugá,^16^ em 2005, apontou percentual dos custos das internações por doenças isquêmicas do coração e por doenças cerebrovasculares, atribuídas ao tabagismo, de aproximadamente 20,0% e 22,0%, respectivamente. No presente estudo, o percentual dos custos das internações para ambas as doenças atribuídas à inatividade física foi de, aproximadamente, 15,0%.

Sichieri et al^18^ analisaram o custo das hospitalizações associadas ao sobrepeso e à obesidade em adultos de 20 a 60 anos em 2001, e observaram que as hospitalizações por diabetes tiveram a maior fração atribuível ao sobrepeso e à obesidade (39,0% e 34,0%, respectivamente). No presente estudo, 26,6% das internações por diabetes foram atribuídas à inatividade física.

Bielemann et al^3^ estimaram em 2007, em Pelotas, RS, a redução dos custos que poderia ser promovida pela atividade física relacionada às internações por doenças do aparelho circulatório e diabetes. Haveria impacto econômico de 50,0% de redução nos custos das internações por doenças do aparelho circulatório e 13,0% das internações por diabetes, caso toda a população se tornasse ativa.

Estudo de Rezende et al^6^ avaliou o impacto da inatividade física na morbimortalidade por quatro doenças crônicas não transmissíveis (doenças cardiovasculares, diabetes tipo 2, câncer de mama, câncer de cólon) no Brasil e observou que a inatividade física foi responsável por 3,0% a 5,0% da incidência dessas doenças e por 5,3% da mortalidade por todas as causas. Tais resultados reforçam o papel da inatividade física como importante fator de risco, impactando significativamente na carga de doenças crônicas.^6^

Estudos documentam impacto econômico significativo da inatividade física nos sistemas de saúde ao redor do mundo. Estudo conduzido no Canadá encontrou que 2,5% do gasto médico total com as doenças crônicas no ano de 1999 foram atribuídos à inatividade física.^10^ Para o Reino Unido, no início da década de 1990, foi estimado que, de 6,5 bilhões de libras gastos no sistema de saúde, 16,0% (1,06 bilhão de libras – aproximadamente R$4,4 bilhões e US$1,7 bilhão) seriam gastos com inatividade física, no início dos anos 2000,^1^ percentual similar ao encontrado no presente estudo. Na China, a inatividade física contribuiu com mais de 15,0% dos gastos médicos e não médicos associados às principais doenças crônicas do país.^20^ Na Austrália, a redução de 10,0% na inatividade física resultaria em menos 6.000 casos de doenças por ano e 2.000 mortes, o que impactaria em grande benefício econômico.^5^

Com a redução de 10,0% na prevalência de inatividade física na população brasileira (de 74,5% para 67,05%), seria obtida redução nos custos de R$24.081.636,89 (US$64.001.766,36) com internações pelas causas estudadas. O benefício econômico seria equivalente à redução de aproximadamente 1,3% do custo total das internações realizadas pelo SUS pelas causas analisadas. Todavia, a comparabilidade de resultados com outros estudos é limitada, uma vez que foram utilizadas diferentes metodologias para estimar o impacto econômico da inatividade física, diferentes populações e períodos, além das diferenças de estrutura dos sistemas de saúde entre os países.

Possível limitação do presente estudo está no fato de que os RR utilizados para estimar a fração atribuível foram retirados da literatura e são oriundos de estudos conduzidos, em sua maioria, em países desenvolvidos com populações distintas da brasileira. A qualidade dos sistemas de notificação do Brasil pode ser outra limitação. Ainda que a qualidade das informações geradas pelo Sistema de Informações Hospitalares tenha melhorado nos últimos anos, a qualidade do sistema está longe do ideal devido à baixa qualidade dos preenchimentos das Autorizações de Internação Hospitalares.^e^ ^,^ ^f^ Em virtude disso, boa parte das internações decorrentes de fraturas osteoporóticas pode ter sido computada como atribuível à causa S72 da CID-10, de fratura do fêmur, o que resulta em subestimativa dos valores apresentados. Entretanto, optou-se por incluir medida mais conservadora, ao invés de analisar as internações cuja causa foi considerada como fratura do fêmur, o que levaria à superestimativa do número de internações e respectivos custos. Por último, a prevalência de inatividade física utilizada (zero min/semana a partir da PNAD) pode ter superestimado os custos atribuíveis à inatividade física por internações no SUS, uma vez que o cálculo da FAI é dependente da prevalência do fator de exposição. Entretanto, houve preferência pela utilização de zero min/semana, visto que a classificação de inatividade física utilizada por diferentes estudos fornece os valores de RR que viabilizam o cálculo da FAI. Isso evita potenciais discrepâncias existentes entre outros possíveis pontos de corte (30 min/semana, e.g.).

Conclui-se que os resultados deste estudo podem contribuir significativamente para conhecer o impacto da inatividade física no sistema de saúde brasileiro. O cálculo da fração atribuível à inatividade segundo sexo e região do País é importante, pois considera a heterogeneidade entre homens e mulheres, além do perfil epidemiológico das regiões, e permite identificar de forma mais fidedigna o impacto da inatividade física às internações realizadas com recursos públicos.

A inatividade física impacta significativamente o número de internações hospitalares pelas doenças crônicas avaliadas e nos custos resultantes, com diferenças na ocorrência das internações e custos onerados pela inatividade dependendo do sexo e região do País. Dessa forma, maior investimento em estratégias de intervenção que visem a redução da inatividade física faz-se necessário, uma vez que contribuiria para redução expressiva dos gastos para o sistema público de saúde e melhoria da qualidade vida e saúde da população brasileira.

## Footnotes

b Instituto Brasileiro de Geografia e Estatística. Pesquisa Nacional por Amostra de Domicílios. Rio de Janeiro (RJ); 2008. [citado 2015 ago 3]. Disponível em: http://www.ibge.gov.br/home/estatistica/populacao/trabalhoerendimento/pnad2008/brasilpnad2008.pdf

c Instituto Brasileiro de Geografia e Estatística. Estimativas populacionais para os municípios brasileiros em 1/7/2012. [citado 2015 ago 3]. Disponível em: http://www.ibge.gov.br/home/estatistica/populacao/estimativa2012/default.shtm

d Valor da conversão em dólar: R$2,6577 de 31 de dezembro de 2014. [citado 2015 ago 03]. Disponível em: http://pt.exchange-rates.org/Rate/USD/BRL/31-12-2014

e Pinto R. Sistemas de informações hospitalares de Brasil, Espanha e Portugal: semelhanças e diferenças [dissertação]. Rio de Janeiro (RJ): Fundação Oswaldo Cruz, Escola Nacional de Saúde Pública Sérgio Arouca; 2010 [citado 2015 abr 29]. Disponível em: http://www.arca.fiocruz.br/bitstream/icict/2292/1/ENSP_Disserta%C3%A7%C3%A3o_Pinto_Rosana.pdf

f Santos A. Sistema de Informações Hospitalares do Sistema Único de Saúde: documentação do sistema para auxiliar o uso das suas informações [dissertação]. Rio de Janeiro (RJ): Fundação Oswaldo Cruz, Escola Nacional de Saúde Pública Sérgio Arouca; 2009 [citado 2015 abr 29]. Disponível em: http://www.arca.fiocruz.br/bitstream/icict/2372/1/ENSP_Disserta%C3%A7%C3%A3o_Santos_Andr%C3%A9ia_Cristina.pdf

## DESTAQUES

As altas taxas de ocorrência de doenças crônicas e os altos custos financeiros decorrentes de seus tratamentos impactam significativamente o sistema público de saúde. O estilo de vida ativo tem potencial para diminuir a ocorrência dessas doenças e, consequentemente, os custos diretos oriundos da utilização do Sistema Único de Saúde (SUS) para seus tratamentos.

Este estudo mostra o impacto econômico do comportamento inativo, ressaltando sua importância na agenda de saúde pública não apenas pelo aspecto econômico, mas principalmente como fator de promoção da saúde e do bem-estar social.

As doenças isquêmicas do coração foram as responsáveis pelos mais altos custos totais e atribuíveis à inatividade física, em todas as regiões e em ambos os sexos, seguidas das doenças cerebrovasculares. Aproximadamente R$276 milhões dos gastos do SUS com estas internações em 2013 foram atribuíveis à inatividade física, o que representou em torno de 15,0% do total de custos.

Os resultados podem fundamentar o planejamento de ações de promoção da saúde à população brasileira que melhorem a situação de doença, a qualidade de vida e o bem-estar social e diminuam os custos do sistema público de saúde com a assistência de alta complexidade.

Rita de Cássia Barradas Barata

Editora Científica
